# A new device for bronchoscopy for better protection

**DOI:** 10.3906/sag-2109-258

**Published:** 2021-12-04

**Authors:** Aslıhan GÜRÜN KAYA, Miraç ÖZ, İncifer KARNAK ORHUN, Serhat EROL, Aydın ÇİLEDAĞ, Demet KARNAK, Akın KAYA

**Affiliations:** 1 Department of Chest Diseases, Faculty of Medicine, Ankara University, Ankara, Turkey; 2 Faculty of Architecture, Gazi University, Ankara, Turkey

**Keywords:** Aerosol spread, bronchoscopy, COVID-19, viral transmission, bronchoscopy cabinet

## Abstract

**Background/aim:**

During the COVID-19 pandemic, the risk of transmission of SARS-CoV-2 has not been precisely known in bronchoscopy procedures. We have designed a cabinet device called Ankara University Bronchoscopy Cabinet (Aubrocab^®^) to protect healthcare. We aimed to evaluate preventing effect of Aubrocab^®^ on aerosol spreading by measuring the particles in the bronchoscopy suite.

**Materials and methods:**

The patients were categorized into two groups as those who underwent bronchoscopy with and without Aubrocab^®^. We measured PM 0.5 levels before and after bronchoscopy in the bronchoscopy suite.

**Results:**

A total of 82 patients, 62 of whom underwent bronchoscopy with Aubrocab^®^, were enrolled in the study. The PM 0.5 level measured before bronchoscopy was similar in both groups, whereas the PM 0.5 level measured after bronchoscopy was lower in the Aubrocab^®^ group (42,603 ± 8,632 vs. 50,377 ± 10,487, p = 0.001). The percent of particle change (50.76 ± 19.91 vs 67.15 ± 24.24, p = 0.003) and the difference of the particle numbers between pre and postprocedure (13,638 ± 4,292 and 19,501 ± 5,891, p < 0.001) were lower in the Aubrocab^®^ group.

**Conclusion:**

Our institution developed a barrier device named Aubrocab^®^ which was shown to prevent excessive aerosol release in addition to routine precautions during bronchoscopy procedures.

## 1. Introduction

Although bronchoscopy is an important tool in both diagnosing and treating lung diseases, the risk of viral spread has raised concerns during the Coronavirus disease-2019 (COVID-19) pandemic. Various guidelines have been published for bronchoscopy applications to reduce the risk of infection transmission in the COVID-19 pandemic. Although these guidelines have similarities and differences, the main recommendations are based on wearing full personal protective equipment, including face shields, gowns, gloves, N-95 masks and respirators or powered air-purifying masks during a bronchoscopy procedure. It has also been suggested that aerosol-generating procedures such as bronchoscopy be applied in negative pressure rooms [1–4]. On the other hand, a negative-pressure room is not always available especially at the bedside or emergent procedures owing to location facilities.

Within the current precautions on bronchoscopy applications, the risk of transmission of severe acute respiratory syndrome coronavirus 2 (SARS-CoV-2) and other respiratory tract pathogens has not already been precisely known. Concerns about the risk of transmission have prompted researchers and clinicians to design new protective devices such as rigid plastic barrier enclosures, widely known as aerosol boxes [5–7]. Although in current forms these devices are thought to be able to form a shield, the effect on their involvement in aerosol spreading is unclear.

Additionally, to our routine personal protective procedures, we have designed a device called Ankara University Bronchoscopy Cabinet (Aubrocab^®^-National trademark registration approval has been obtained for this name) to protect the healthcare team, patient, or area of operation from droplets. Given that The Centers for Disease Control and Prevention (CDC) indicated airborne transmission via small droplets and particles as an important route of virus spread in the pandemic^1^ we aimed to evaluate preventing effect of Aubrocab^®^ on aerosol spreading by measuring the number of particles in the bronchoscopy suite.

## 2. Materials and Methods

### 2.1. Study design

The prospective study was conducted in a bronchoscopy unit of a university hospital to evaluate the efficacy and safety of bronchoscopy with and without newly invented bronchoscopy cabin Aubrocab^®^. In our preliminary measurements by 6 bronchoscopies each (total 12) with or without Aubrocab^®^ we observed 41% and 77% of particle increase in the bronchoscopy suite after the procedure, respectively.

With the prediction that the rate of particle increases in procedures with Aubrocab^®^ will be significantly lower than the procedures without it. The estimated required sample size was a total of 80 patients, 60 with Aubrocab^®^ and 20 without Aubrocab^®^, who were included in the study with 80% power at d = 0.50 effect size, α = 0.05 error level and an allocation ratio of 3:1. The sample size for the study was calculated using the GPower program (GPower Version 3.1.9.2). Patients were randomly assigned in a 3:1 ratio to the procedure with or without Aubrocab®.

The study protocol was approved by the Ethics Committee of the Ankara University School of Medicine on 09 July 2020 (approval No. I7-400-20). Written informed consent was obtained from each of the patients before the procedure, and the study was conducted in accordance with the amended Declaration of Helsinki.

### 2.2. Study patients

All patients were recruited from the bronchoscopy unit of the university hospital from 01.08.2020 to 30.04.2021. All bronchoscopy decisions were made by an experienced pulmonologist. In line with our standard of the fiberoptic bronchoscope (FB) procedure, all patients were evaluated by clinical and laboratory findings. Absolute contraindications for the FB procedure are: PaO_2_ < 60 mmHg after 100% oxygen administration, presence of bronchospasm, respiratory acidosis, cardiovascular system diseases (recent myocardial infarction, stable-unstable angina, arrhythmia, hypertension), presence of cerebrovascular pathology, increased intracranial pressure, convulsion, pneumothorax, bleeding diathesis (international normalised ratio (INR) ≥ 1.5), thrombocytopenia (<50,000/mm3), thrombocyte dysfunction, severe anaemia (Hb < 8g/dL), portal hypertension, and uremia. Patients were also evaluated for possible signs of pulmonary infection. The following patients were excluded from the study: history of fever in the last 14 days, history of contact with a person diagnosed with COVID-19 within 14 days, subjects with newly developed cough, sputum, shortness of breath, diarrhea findings within 14 days and patients whose radiologically findings suggestive of viral infection. Patients with these findings were referred to relevant clinics to be investigated for possible infection conditions, and bronchoscopy procedures were postponed. Within 24 h before the bronchoscopy procedure, the SARS-CoV-2 reverse transcriptase-polymerase chain reaction (rt-PCR) test was performed, when the test was negative.

### 2.3. Bronchoscopy procedure

Conventional procedures were performed in the supine position via the transoral route by trained bronchoscopists (DK, AÇ, AGK, MÖ, SE, AK) using a flexible bronchoscope (FB) (Olympus Video bronchoscope BF1T200; Olympus, Tokyo, Japan). Endobronchial ultrasound-guided transbronchial needle aspiration(s) (EBUS-TBNAs) were performed using a bronchoscope (BF-UC180F; Olympus Medical, Japan) with an electronic convex-array ultrasound processor (EU-ME1; Olympus Medical, Japan) to the distal tip. Bronchoscopy suite staff was not more than four, all of whom had personal protective equipment during the procedure. These procedures were done under local anaesthesia with 2% lidocaine solution for cough suppression with or without sedation with midazolam (0.01–0.1 mg/kg) and/or fentanyl (25–100 μg). Additionally, 2–4 mL of 2% lidocaine was administered via a bronchoscope during the procedure at the vocal cord level as a local anaesthetic for cough suppression.

### 2.4. Cabinet system (Aubrocab^®^)

**Figure 1:**
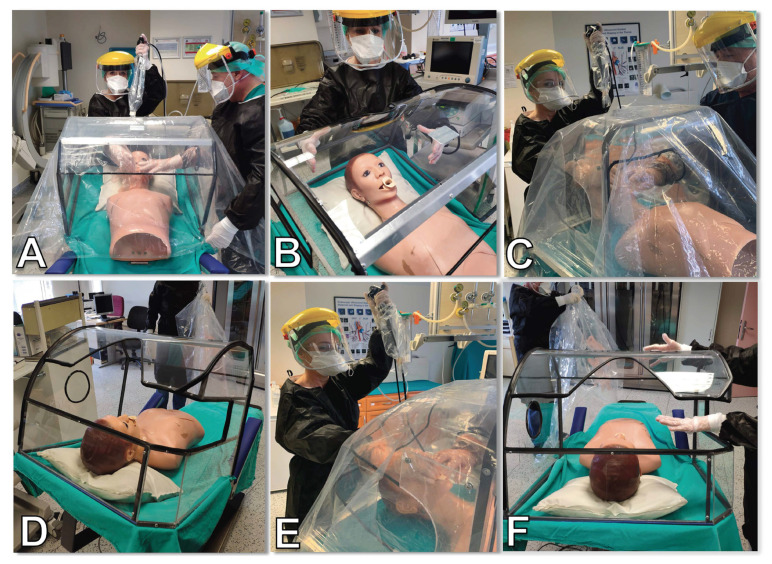
The front view of Aubrocab^®^ during the procedure (A), the body of the cabinet, which is made of plexiglass and stainless steel (B), the transparent polyethylene cover on the body and polyethylene gloves are worn by the bronchoscopist and assisting staff (C), right side rear oblique view of the body of the cabinet and its holes (D), right side front oblique view (E) and removal of polyethylene cover after the procedure (F).

**Figure 2:**
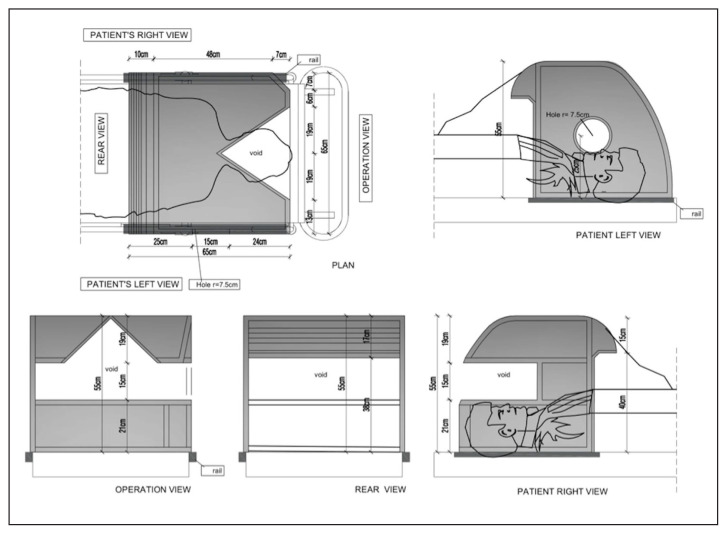
The plan of the Aubrocab^®^ from different views.

A bronchoscopy cabinet with the name of Aubrocab^®^ has been designed for the healthcare team during the bronchoscopy procedures in our clinic to reduce the risk of transmission. Aubrocab^®^ is a cabinet system consisting of hygienic material such as plexiglass, stainless-steel and rubber, with a smooth surface that prevents microorganisms from adhering and can be easily washed. An antibacterial transparent polyethylene cover is placed on the whole surface of the Aubrocab^®^. This transparent cover creates a barrier between the patient and the healthcare personnel by covering both surface and apertures of the cabinet. Two adapted polyethylene gloves, corresponding to the holes on the surface of Aubrocab^®^, can be easily worn and taken off by the bronchoscopist and assisting staff who holds the mouthpiece and oxygen mask during the procedure. FB is also covered by camera coverage beginning from the suction canal to the upper half. The Aubrocab^®^ is represented in Figure 1: the front view during the procedure (Figure 1A), the body of the cabinet, which is made of plexiglass and stainless steel (Figure 1B), the transparent polyethylene cover on the body and polyethylene gloves are worn by the bronchoscopist and assisting staff (Figure 1C), right side rear oblique view of the body of the cabinet and its holes (Figure 1D), right side front oblique view (Figure 1E) and removal of polyethylene cover after the FB (Figure 1F). Also, the plan of Aubrocab^®^ from a different view is given in Figure 2. After each procedure, Aubrocab^®^ is disinfected as the following steps: 1) After waiting for five min, polyethylene cover is folded inwards and destroyed as medical waste; 2) the patient is removed from the cabin by wearing a mask; 3) the surface of the cabinet is wiped down for complete removal of mucus, blood, and visible secretions via disposable towels inside the procedure room; 4) the cabinet is mechanically washed cleaned with a neutral detergent followed by decontamination of surfaces using a high-level disinfectant. Staff should wear PPE during all of the disinfection processes; 5) Terminal decontamination of bronchoscopy suit using a mobile automated UV light unit.

### 2.5. Particle measurements (PM)

The 42 square m area bronchoscopy suite where the study was conducted is ventilated by a HEPA filter cleaning PM over 0.5μm. The airborne particles measurements were performed 5 m away from the door and near the air conditioner by the same researcher at pre and postprocedural time immediately via “Particles Plus^®^ 8306 particle measurement device” that counts have a detectable minimum particle diameter of 0.3 μm. In this study, the concentrations of a PM recorded are the cumulative data, and PM 0.5 are the concentrations of particles ≤ 0.5μm. Such particles (<0.5 μm) may reach and deposit in the alveoli where the air velocity is low and may cause infection. This accumulation is directly proportional to the procedure time [8]. The range of 2.5–10 μm, including 0.5μm particles, are called coarse particles and they are known to be very dispersive [9]. After measuring the baseline and after procedure PMs, the percentage of particle number change was calculated as follows: (post-pre procedural number/preprocedural number) × 100 and given as percent particle change.

### 2.6. Statistical analyses

The data was analysed using IBM SPSS Statistics (Version 22.0. Armonk, NY: IBM Corp.). Continuous variables with normal distribution were presented as mean ± standard deviation and as median [25th–75th percentiles, interquartile range (IQR)] for nonnormal distributed variables. A Kolmogorov-Smirnov test was used to analyse the distribution of variables, and a Levene test was applied to assess the equality of variances. An unpaired student’s t-test or a Mann-Whitney U test were used to compare the two groups. Categorical data were expressed as numbers and percentages and compared with a chi-square test or Fisher’s exact test, as appropriate. Repeated-measures analysis of variance (ANOVA) model was used to analyse the measurements taken from a homogeneous group of patients at different time points. The statistical significance level was expressed as p < 0.05 for all tests.

## 3. Results

Eighty-two patients enrolled in the study underwent bronchoscopy. The mean age of the study group was 59.8 ± 12.8 years. Among the study patients, 53 (64.6%) were male, and 62.2% of them had at least one comorbid disease. In 82 bronchoscopy procedures, bronchial washing samples (n = 42), bronchial biopsy (n = 21), transbronchial needle aspiration (n = 40), bronchial brushing (n = 8), bronchoalveolar lavage samples (n = 13), and transbronchial biopsy (n = 3), were obtained.

While 60 procedures (73.2%) were performed under moderate sedation using fentanyl and/or midazolam, 22 were performed under local anaesthesia. Forty (48.8%) of 82 bronchoscopy procedures were performed with endobronchial ultrasound (EBUS). While 62 (75.6%) of bronchoscopies were performed with the Aubrocab^®^ and 20 (37.8%) patients were performed without this system. Basal demographic and clinical features of the groups that underwent bronchoscopy with and without Aubrocab^®^ were similar (Table).

**Figure 3:**
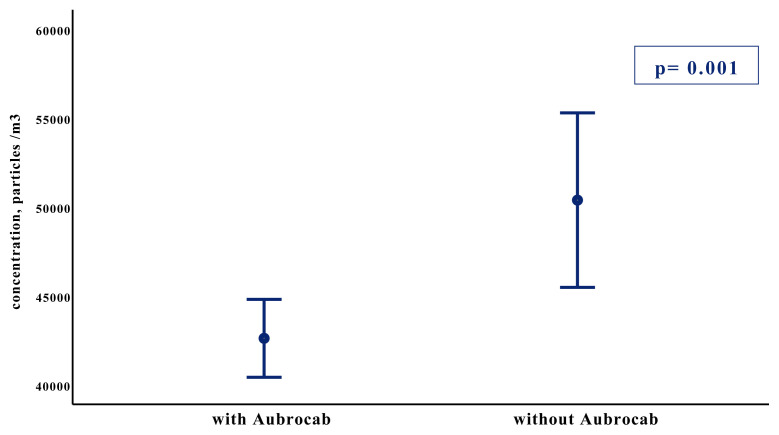
Measured PM 0.5 levels of bronchoscopy room air at the postprocedural time with and without Aubrocab^®^ (p = 0.001). Y-axis represents the mean and 95% confidence interval of particles count. The X-axis represents the two groups of patients.

**Figure 4:**
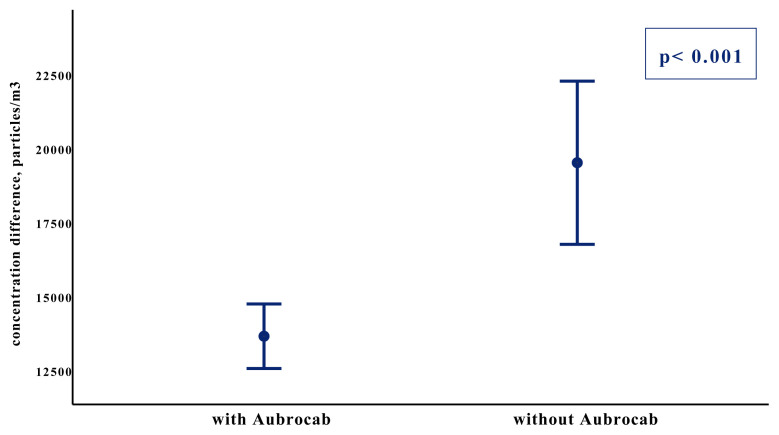
Pre and postprocedural particle difference in number with and without Aubrocab^®^ (p < 0.001). Y-axis represents the mean and 95% confidence interval of the difference between pre and postprocedural particles in number. The X-axis represents the two groups of patients.

Airborne particle concentration level of PM 0.5 measured before bronchoscopy were similar in both groups (28,965 ± 8,907 vs. 30,875 ± 8,470, p = 0.393), whereas the PM 0.5 level measured just after bronchoscopy was significantly lower in the Aubrocab^®^ group compared to the group without Aubrocab^®^ (42,603 ± 8,632 vs. 50,377 ± 10,487, p = 0.001) (Figure 3). The difference of the particle numbers between pre and postprocedure were also measured and compared in groups with or without Aubrocab^®^ 13.638 ± 4.292 and 19.501 ± 5.891 showing statistical significance (p < 0.001) (Figure 4).

**Figure 5:**
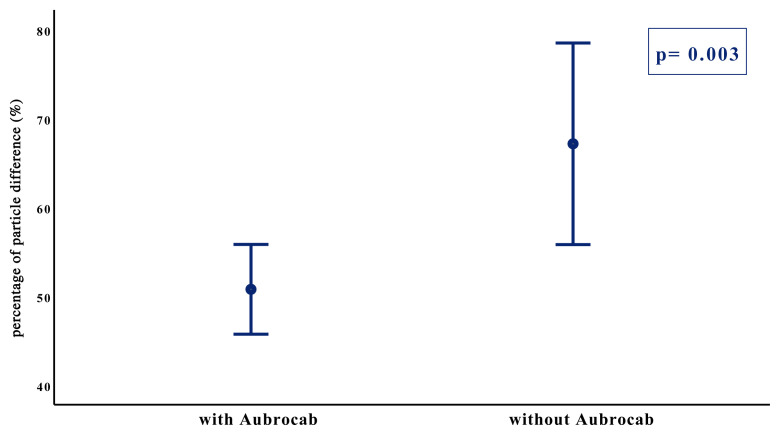
Pre and postprocedural particle change in percentage with and without Aubrocab^®^ (p = 0.003). Y-axis represents the mean and 95% confidence interval of percentages of particles change. The X-axis represents the two groups of patients.

The percent particle changes between baseline and after bronchoscopy measurements of particles were compared between the groups. The analyses showed that the percent particle change was significantly lower in the Aubrocab^®^ group (50,76 ± 19,91 vs. 67,15 ± 24,24, p = 0.003) (Figure 5).

**Figure 6:**
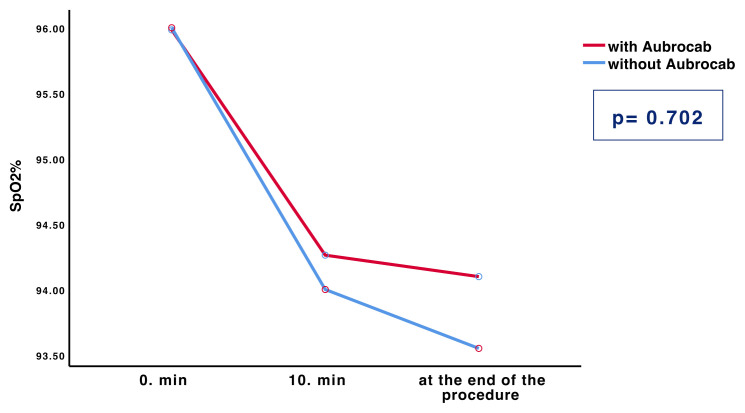
A repeated-measures ANOVA revealed no significant difference in treatment between procedures performed with and without Aubrocab^®^ (p = 0.702). Y-axis represents the SpO_2_ level ( % ). The X-axis represents the time.

All bronchoscopy procedures were well tolerated; no hemodynamic instability occurred during the procedure, and no patient required endotracheal intubation or escalation for respiratory support. None of the patients revealed symptoms suggestive of hypercapnia during or after bronchoscopy. ANOVA model for repeated measurements revealed no differences in the change in oxygen saturation measured by pulse oximetry (SpO_2_) by the time between procedures which were performed with and without Aubrocab® (p = 0.702) (Figure 6). Although the SpO_2_ value decreased compared to the baseline measured value during the bronchoscopy procedures in both groups, this decreasing trend was not clinically significant.

## 4. Discussion

This prospective study revealed that the use of Aubrocab^®^, a bronchoscopy cabinet designed in our clinic, may be an effective method to reduce aerosol dispersion during the bronchoscopy procedure. During the COVID-19 pandemic, occupational exposure, and increased risk of infection in healthcare workers remain a major concern worldwide. To protect healthcare staff in the bronchoscopy suite and patients and next coming patients undergoing aerosol-generating procedures such as tracheal intubation and bronchoscopy, various containment boxes, cabinets or tents have been designed to act as a barrier against the spread of aerosols [10–14]. However, there is no data about the protective strength of the devices from aerosolised pathogens [12]. To the best of our knowledge, this is the first study to evaluate changing particle count during the procedure in a bronchoscopy suite without negative pressure.

Canelli et al. reported a simulation study that exploded a small latex balloon containing fluorescent dye placed in the hypopharynx of a human-like model. The explosion of the balloon represented a crude depiction of a cough, and the authors repeated the experiment with and without the aerosol box to illuminate the scene with ultraviolet light to visualise the spreading of the dye in each situation. Their simulation suggested that the box helps restrict the spread of droplets and may a barrier enclosure provide a modicum of additional protection and could be considered an adjunct to standard personal protective equipment [7]. Similarly, Kloka and colleagues conducted another model simulation study in which the fluorescent dye represented droplets and aerosols with another device named COVid aErosol pRotEction Dome - COVERED. The authors suggested the protective effect of COVERED against coughing during intubation evaluated of visible with fluorescent dye [15]. Recker and Gross described a protective bronchoscopy tent. The authors reported that they did not detect any droplet leakage during simulated aerosol generation using a standard nebuliser with backlighting to highlight particles [11]. Both studies showed that with a fluorescent dye, the dye particles representing the droplets are large enough to be visualised and can be delineated of large droplets. However, the visible fluorescent dye could not simulate the small size aerosols that could reach into the lung parenchyma via inhalation. Doggett et al. noted an increase in fine particles during elective bronchoscopy procedure, whereas noted a reduction of larger particle generation. The authors attributed this reduction of larger particles to obstructions such as the inserted bronchoscope and gauze used around the scope and bite block, which may affect releasing larger particles during procedures [16].

In the present study, we evaluated the effectivity of the Aubrocab^®^, via measuring a small particle size of less than 0.5 μm may diffuse and accumulate in the alveoli because of smaller airway structures and longer residence time [17,18]. Our findings suggested that the use of Aubrocab^®^ can decrease the small aerosol particles spread during bronchoscopy. However, it should be noted that the number of particles does not reflect the actual quantity of the virus-containing particles. Previous studies showed viral infections agents could be carried by aerosols, especially in the range of very small size, and measurement of those can be used to simulate viral spread [19]. Guzman conducted a study to evaluate the effect of bioaerosol size in SARS-CoV-2 transmission and reported maximum concentrations of viral RNA with 40 copies per cubic m in particles with sizes from 0.25 to 0.5 μm [20]. Similarly, in another study, peak concentrations of SARS-CoV-2 RNA 40 and 9 copies/m^3^ in the aerosol with sizes of 0.25–0.5 μm and 0.5–1.0 μm, respectively were detected [21]. Lednicky et al. observed viable SARS-CoV-2 RNA in the collection of airborne particles of the size range of 0.25 to 0.5 μm from the air of a car being driven by a person with COVID-19 [22]. Previously Hersen and colleagues reported that patients with respiratory system infectious, particularly influenza and corona infections produce many aerosols, especially in the range of small size with less than 1 μm [23]. Many other studies revealed a significant amount of influenza virus in the aerosol with small size [24–26].

Although Aubrocab^®^ has similarities with previously designed devices for barrier enclosure to aerosol [6, 12, 13, 15]; probably had more advantages in that it is ergonomically designed with its curve shape that provides bronchoscopist can stand closer to the patient and allows for comfortable movement of the hands. Also, the device provides patient visibility and communication with its colourless and transparent structure. Polyethylene cover that placed the whole surface and polyethylene gloves positioned to an aperture through which the bronchoscopists’ hands are passed act a shield against aerosol spreading. Aubrocab^®^ is also more practical and economical than devices with negative pressure or smoke evacuation attachments [27, 28].

We did not observe any major complications requiring early termination of the procedure, endotracheal intubation, hemodynamic instability, or escalation in the respiratory support in patients who underwent a bronchoscopy with or without Aubrocab^®^. No significant decrease in oxygen saturation was detected in the patients monitored during the procedure. In addition, there was no hypercapnia symptom that may be associated with hypoventilation or rebreathing that might occur during the procedure [29]. We suggest that the appropriate patient evaluation before the procedure is the key factor to reduce complication risk for all bronchoscopy procedures. Such closed systems like Aubrocab^®^ may trigger claustrophobia, and anxiety [12], however, we did not observe any. We believe that good communication and appropriate sedation are essential for success.

One of the limitations of our study is that it is carried out at a single medical institution. Besides, conditions such as coughing and sneezing, which increase the aerosol formation have not been recorded, which may have affected the particle measurements in the environment. Moreover, we only measured the number of particles yet we do not know whether these particles contain infected material or not. Therefore, it may not be possible to reach a clear conclusion by measuring the number of particles in terms of viral transmission. In the preliminary phase of the study, we measured significantly fewer particles in the environment by using Aubrocab^®^. With this knowledge, on further study, our colleagues refused to perform bronchoscopy without Aubrocab^®^ in order to increase the control group. Therefore, we have a low number of the control group.

In conclusion, our institution developed an ergonomic and reusable barrier device named Aubrocab^®^ which was shown to prevent excessive aerosol release in addition to routine precautions and standard personal protective equipment during bronchoscopy procedures.

## Figures and Tables

**Table:** Features of the patients included in the study.

	With Aubrocab^®^ (n = 62)	Without Aubrocab^®^ (n = 20)	*p*
Age (years)	58.5 ± 12.8	63.9 ± 12.3	*0.103*
Gender (male)	41 (66.1%)	12 (60%)	*0.618*
Any comorbid disease	36 (58.1%)	15 (75%)	*0.174*
Chronic pulmonary disease	16 (25.8%)	6 (30%)	*0.713*
Hypertension	15 (24.2%)	7 (35%)	*0.343*
Diabetes mellitus	6 (9.7%)	5 (26.3%)	*0.118*
Coronary artery disease	5 (8.1%)	4 (20%)	*0.211*
Malignancy	11 (17.7%)	4 (20%)	*0.999*
Bronchoscopy	32 (51.6%)	10 (50%)	*0.999*
Bronchoscopy with EBUS	30 (48.4%)	10 (50%)	
Local anaesthesia without sedation	19 (30.6%)	3 (15%)	*0.170*
Local anaesthesia with sedation	43 (69.4%)	17 (85%)	
Duration of procedure (min)	19 (IQR_25-75_ 17–26)	25.5 (IQR_25-75_ 18–31)	*0.113*

## Footnotes

1 CDC (2020) Scientific Brief: SARS-CoV-2 and Potential Airborne Transmission [online]. Website https://www.cdc.gov/coronavirus/2019-ncov/science/science-briefs/sars-cov-2-transmission.html?CDC_AA_refVal=https%3A%2F%2Fwww.cdc.gov%2Fcoronavirus%2F2019-ncov%2Fscience%2Fscience-briefs%2Fscientific-brief-sars-cov-2.html (Accessed 19 May 2021),
